# Investigation of the effect of Myricetin on Cisplatin-induced liver hepatotoxicity

**DOI:** 10.1590/1806-9282.20240136

**Published:** 2024-07-19

**Authors:** Sümeyye Aksoy, Nurhan Kuloğlu, Derya Karabulut, Birkan Yakan

**Affiliations:** 1 Erciyes University, Institute of Health Sciences – Kayseri, Turkey.; 2 Niğde Ömer Halisdemir University, Department of Healthcare Services – Niğde, Turkey.; 3 Erciyes University, Faculty of Medicine, Department of Histology and Embryology – Kayseri, Turkey.

**Keywords:** Antioxidant, Cisplatin, Myricetin, Hepatotoxicity, Rat

## Abstract

**OBJECTIVE:**

Cisplatin, a widely used anticancer agent, induces hepatotoxicity alongside organ damage. Understanding Cisplatin's toxicity mechanism and developing preventive measures are crucial. Our study explores Myricetin, a flavonoid, for its protective effects against Cisplatin-induced hepatotoxicity.

**METHODS:**

In our study, a total of 32 Wistar albino male rats were utilized, which were categorized into four distinct groups: Control, Myricetin, Cisplatin, and Myricetin+Cisplatin. For the histological assessment of hepatic tissues, hematoxylin–eosin and periodic acid Schiff staining were employed, alongside immunohistochemical measurements of TNF-α, interleukin-17, and interleukin-6 immunoreactivity. Additionally, aspartate transaminase and alanine transaminase values were examined by biochemical analysis.

**RESULTS:**

In the histological evaluation of the tissues, a normal healthy cell structure and a strong periodic acid Schiff (+) reaction were observed in the hepatocyte cells in the tissues of the Control and Myricetin groups, while intense eosinophilia, minimal vacuolization, congestion, and sinusoidal expansions were observed in the hematoxylin–eosin stainings, and a decrease in the positive reaction in the periodic acid Schiff staining was observed in the Cisplatin group. Consistent with these histological findings, an increase in TNF-α, interleukin-17, and interleukin-6 expressions (p<0.0001) and a concomitant increase in aspartate transaminase and alanine transaminase values were observed in the Cisplatin group. In the group protected by Myricetin, a significant improvement was observed in all these histological and biochemical values.

**CONCLUSION:**

Cisplatin induces notable histopathological alterations in the liver. In this context, Myricetin exhibits the potential to alleviate Cisplatin-induced damage by modulating histological parameters and biochemical processes.

## INTRODUCTION

Cisplatin (Cis) is a derivative of platinum salts and is a chemotherapy drug used to inhibit the growth of cancer cells^1^. Cis, a teratogenic, mutagenic, and carcinogenic effective agent, is used in various cancer treatments, such as ovarian, cervix, and head and neck cancer^2^. In addition to its anti-tumoral effects, Cis causes many undesirable effects, such as hepatotoxicity^1-3^.

Myricetin (Myr) is a member of the flavonoid group called flavonols. It is obtained from various fruits, vegetables, tea, berries, and similar plants. Myr has been found to have anti-proliferative and anti-angiogenic effects in many types of cancer^4^. Myr is reported to be effective in many diseases, including different types of tumors, inflammatory diseases, atherosclerosis, thrombosis, cerebral ischemia, diabetes, Alzheimer's disease, and pathological microbial infections^5^. Myr has demonstrated therapeutic potential in reducing alcohol-induced liver damage, indicating its effectiveness in alleviating hepatic injury. It may serve as a specific protective agent against liver damage^6^. Studies conducted with Myr suggest that it reduces liver DNA damage induced by chemical substances and reduces increased serum levels of aspartate transaminase (AST), alanine transaminase (ALT), alkaline phosphatase (ALP), and total bilirubin^7^.

In response to liver damage, specific intracellular processes are initiated to maintain liver integrity. TNF is the main mediator of these processes and activates different cellular responses such as proliferation, survival, and death^8^. TNF-α cooperates with interleukin-17 (IL-17) to synergistically induce a massive production of interleukin-6 (IL-6) and interleukin-8 (IL-8) by endothelial cells, skin and synovial fibroblasts, and hepatocytes^9^.

Our study aimed to investigate the potential treatment effects of Myr, a natural ingredient, to alleviate liver damage caused by Cis and offer a protective approach. Additionally, in this study, we aimed to elucidate the effects of these cytokines on liver damage and Myr treatment by evaluating the immunoreactivity of pro-inflammatory cytokines TNF-α, IL-17, and IL-6.

This important study may contribute to the understanding of a potential new approach to reduce liver toxicity associated with cancer treatment and may help develop better treatment strategies in the future.

## METHODS

### Animals

In this study, conducted at the Department of Histology and Embryology, Erciyes University in Kayseri, Turkey, a total of 32 male Wistar albino rats, aged 9 weeks and weighing between 220 and 240 g, were utilized. These rats were procured from the Erciyes University Experimental and Clinical Research Center. The rats were housed in standard cages, maintained at a temperature of 21°C, and subjected to a 12-h light/dark cycle, with their nutritional and hydration requirements met. Prior to the study, the rats were individually weighed, and animals of similar weight were grouped. Ethical considerations and established guidelines for the care and well-being of all animals were strictly adhered to throughout the study.

### Experimental design

The rats were randomly assigned to four groups of eight. Control group: rats that had access only to water and food throughout the experiment; Cis group: a single dose (7.5 mg/kg) of Cis was administered intraperitoneally on the seventh day^10^; Myr group: Myr (10 mg/kg) was administered intraperitoneally for 7 days^11^; Myr+Cis group: Myr (10 mg/kg) was administered intraperitoneally for 7 days, and at the end of the seventh day, a single intraperitoneal dose of Cis (7.5 mg/kg) was given. After the experimental procedure, the rats were anesthetized and then sacrificed.

### Chemicals

Cisplatin (Koçak Farma, Istanbul, Turkey) was used intraperitoneally as an inducer of liver damage. Myr (Sigma-Aldrich, St. Gallen, Switzerland) was used as a protective and therapeutic substance in the experiment.

### Histological examination

At the end of the experiment, rats were anesthetized using anesthetic agents [ketamine (75 mg/kg)+xylazine (10 mg/kg)]. Liver tissues were fixed in a 4% formaldehyde solution. Then, the routine light microscopic procedure was applied. For this procedure, dehydration was first applied to the tissues. Then, it was made transparent by holding it in xylene, and fixed blocks were made with paraffin. Sections were taken from paraffin blocks and stained with hematoxylin–eosin and periodic acid-Schiff (PAS). Sections were examined under a light microscope^12^.

To determine the changes occurring as a result of damage to the liver tissue, the immunohistochemical staining method was applied to show the expressions of TNF-α, IL-17, and IL-6^12^.

### Biochemical analysis

Alanine aminotransferase and AST values of blood serum samples taken at the end of the experiment were analyzed by the service in the Erciyes University Central Biochemistry Laboratory.

## RESULTS

### Histopathological findings

**Figure 1:**
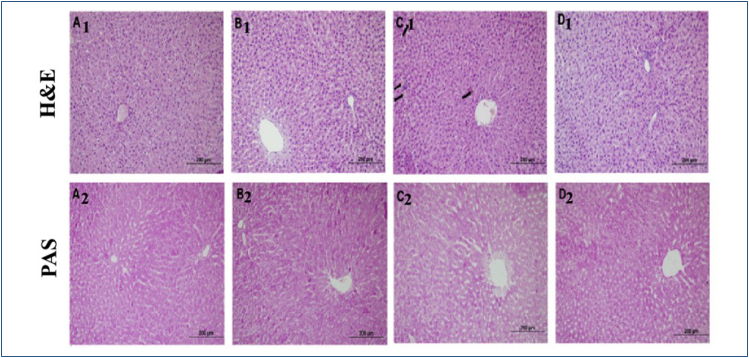
Hematoxylin–eosin (H&E) staining of liver tissue of experimental groups. A_1_: Control group, B_1_: Myricetin group, C_1_: Cisplatin group, D_1_: Cisplatin+Myricetin group. Black arrows: It shows areas with high eosinophilic staining in hepatocyte cells. Liver tissue periodic acid Schiff (PAS) staining of the experimental groups. A_2_: Control group, B_2_: Myricetin group, C_2_: Cisplatin group, D_2_: Cisplatin+Myricetin group. 20× objective, scale bar 200 μm.

The histological structure of normal healthy cells was observed in the liver sections of the control and Myr groups. It is seen that some of the hepatocytes in the Cis-treated group have more intense eosinophilic staining. It is seen that there is irregularity in the arrangement of the cell cords and widening and distortions in the sinusoidal spaces in some sections. However, areas of congestion and mononuclear cell infiltration were detected in the tissues belonging to the damage group. In addition, in the liver tissues of the Myr+Cis-applied group, there was a decrease in eosinophilic staining compared to the damage group, the hepatocyte arrangement around the central vein was more regular, and the widening in the sinusoidal spaces decreased (Figure 1).

Periodic acid Schiff staining was performed to evaluate the glycogen content of liver tissues. In the sections of the control and Myr groups, it was observed that hepatocyte cells gave a strong PAS-positive reaction. However, in the Cis-applied group, there was a decrease in PAS positivity compared to the control group. In addition, an increase in PAS positivity density was observed in the Myr+Cis-applied group (Figure 1).

### Immunohistochemical findings

**Figure 2:**
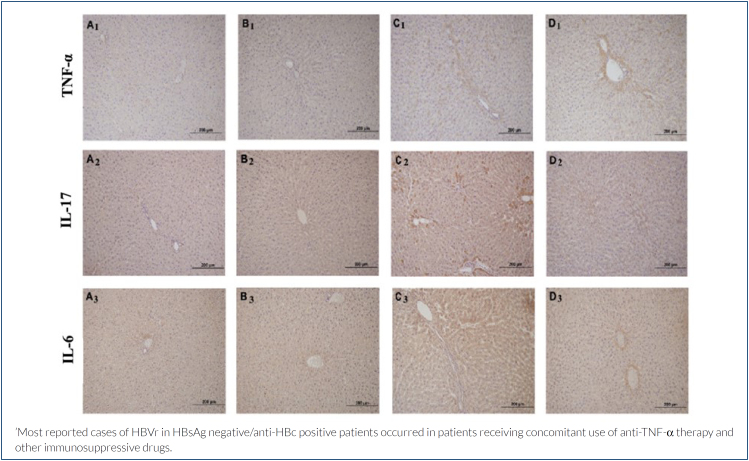
TNF-α immunohistochemistry staining image A_1_: Control group, B_1_: Myricetin group, C_1_: Cisplatin group, D_1_: Cisplatin+Myricetin group. IL-17 immunohistochemistry staining image. A_2_: Control group, B_2_: Myricetin group, C_2_: Cisplatin group, D_2_: Cisplatin+Myricetin group. IL-6 immunohistochemistry staining image. A_3_: Control group, B_3_: Myricetin group, C_3_: Cisplatin group, D_3_: Cisplatin+Myricetin group. 20× objective, scale bar 200 μm.

In the study, immunohistochemical staining was performed to determine the TNF-α, IL-17, and IL-6 immunoreactivity of the experimental groups. When TNF-α protein expression was examined, a significant increase was observed in the Cis group applied alone compared to the other groups, while this increase was observed to be minimally reduced in Cis applied together with Myr. Similarly, a significant increase in IL-17 and IL-6 expression was observed in the Cis group administered alone, while a statistically significant improvement was observed in the Cis group administered together with Myr (Figure 2 and Table 1).

**Table 1:** Liver tissue TNF-α, interleukin-17, and interleukin-6 immunoreactivity measurement results and serum aspartate transaminase and alanine transaminase results of the experimental groups.

Groups	Control	Myr	Cis	Myr+Cis	p
TNF-**α**	74.7±2.^4^a	79.4±2.5^b^	80.8±5.5^cd^	79.9±3.5^bd^	<0.0001
IL-17	88.2±4.9^a^	88.7±5.8^a^	95.1±6.1^b^	91.4±3.9^c^	<0.0001
IL-6	81.8±2.2^a^	86.3± 4.7^b^	90.8±9.7^c^	85.1±2.8^bd^	<0.0001
**Groups**	**Control**	**Myr**	**Cis**	**Myr+Cis**	**p**
AST	80.8±10.3^a^	68.6±16.1^ab^	87.1±10.6^ac^	84.6±6.3^a^	0.0503
ALT	45.0±4.0^a^	40.3±4.8^a^	47.3±5.5^ab^	39.1±2.9^ac^	0.0143

IL-17: interleukin-17; IL-6: interleukin-6; AST: aspartate transaminase; ALT: alanine transaminase; Myr: Myricetin; Cis: Cisplatin. Data are expressed as mean±standard deviation. There is no significant difference between groups containing the same letter (a–d). p<0.05 was considered significant.

### Biochemical fındings

While minimal changes were observed between the groups in AST values, in the comparison of the Cis group applied alone and the Cis group applied together with Myr in ALT values, it was seen that Myr corrected the increase in the damage group statistically significantly (Table 1).

### Statistical analyses

In the study, statistical analysis of the results obtained from biochemical and immunoreactivity data was performed using GraphPad (Prism 8.00 for Mac, GraphPad Software, La Jolla, California, USA). The D'Agostino Pearson omnibus test was used to check the normal distribution of the data. Data were expressed as mean±SD and analyzed by one-way ANOVA test and Tukey's post-hoc test for parametric tests. p<0.05 was considered significant in the analysis.

## DISCUSSION

It is known that Cis causes damage to many tissues, and one of these negative effects is liver hepatotoxicity^13,14^. Cis causes morphological changes in the arrangement of hepatocyte cords^15^. For example, in the liver, irregularity in the hepatic cords, portal triad fusion and central vein obstruction^16^, degenerative hepatocytes^14^, pyknosis of hepatocyte nuclei around the vena centralis in some and hypertrophy, inflammation, hypertrophy in some hepatocytes, vascular occlusion, sinusoidal dilatation^17^, and congestions are a few of them^18^.

Similar to the results of these studies, according to our histological data, in the liver sections of rats administered Cis alone, compared to the control group, there were changes in the classical lobule structure, intense eosinophilia in hepatocytes, thickening of the vena centralis wall, minimal vacuolar changes in the cytoplasm, sparse mononuclear cell infiltration, and enlargements of the sinusoids. It was determined that the strong PAS-positive reaction seen in the control group decreased in the damage group. This decrease in PAS positivity may be due to damaged mitochondria and decreased glucose levels. Histological data of the Cis group applied together with Myr show that cellular deteriorations were improved and there was an increase in the PAS-positive reaction in hepatocytes compared to the damage group. After Cis administration, significant glycogen loss is observed in hepatocytes. Myr prevents this glycogen loss. Glycogen positivity in hepatocytes is confirmed by amylase incubation, which abrogates the PAS reaction in these compartments^15^.

Single-dose Cis administration increases AST, ALT, and ALP activities^19^ and causes a significant increase in serum TNF-α levels compared to the control group^16,20^. Our data in our study increased the serum AST and ALT values of the group administered a single dose of Cis, similar to the literature. However, a significant improvement was observed in liver enzyme values, especially ALT values, in the group protected by Myr against Cis-induced damage.

Similarly, in Cis-induced damage studies, severe TNF-α expressions in the Cis group^3^ and an increase in oxidant parameters, a decrease in antioxidant parameters, and a severe increase in TNF-α and Caspase-3 expressions in immunohistochemical evaluations were noted^21^. Our findings in our study are similarly manifested by the upregulation of TNF-α, IL-17, and IL-6 in the damage group. In hepatotoxicity, Myr prevents hepatotoxicity by modulating the production of free radicals and inflammatory markers. Additionally, Myr treatment reduced hepatotoxicity and ethanol-induced inflammatory markers such as IL-6^6^. Apart from this, hemorrhagic necrosis of liver tissues in hepatotoxicity and inflammatory cell infiltration in the portal area were significantly reduced by Myr pretreatment, resulting in less bleeding and cell infiltration, indicating that Myr has a protective effect on liver tissues^22^. In our current study, although the histological disorders and biochemical changes occurring in the injury group showed a partial improvement in TNF-α expression in the immunohistochemical values of the Myr group applied for protective purposes along with Cis, a significant improvement was observed in IL-17 and IL-6 protein expressions. Biochemical values similarly support these findings.

## CONCLUSION

The decrease in histological damage markers and biochemical activities of Myr against Cis-induced hepatotoxicity unequivocally demonstrates its protective effect on cellular structure, highlighting the need to enhance the dose and duration of Myr application to optimize its effectiveness, which constitutes a crucial avenue for further research.

## ETHICS COMMITTEE APPROVAL

All procedures were carried out with the approval of the Ethical Committee (date 2021, decision no: 21/187 and date 2023 23/052) of Erciyes University Experimental Animals.
